# Implant Navigation During TMJ Reconstruction: A Proof-of-Concept Study

**DOI:** 10.3390/jpm16020122

**Published:** 2026-02-18

**Authors:** Lauren C. M. Bulthuis, Jean-Pierre T. F. Ho, Petra C. M. Zuurbier, Michail Koutris, Ruud Schreurs, Jan de Lange

**Affiliations:** 1 Department of Oral and Maxillofacial Surgery, Amsterdam UMC, University of Amsterdam, Meibergdreef 9, 1105 AZ Amsterdam, The Netherlands; 2 Academic Centre for Dentistry Amsterdam (ACTA), University of Amsterdam and Vrije Universiteit Amsterdam, 1081 LA Amsterdam, The Netherlands; 3 Department of Oral and Maxillofacial Surgery, Northwest Clinics, 1815 JD Alkmaar, The Netherlands; 4 Department of Orthodontics, Academic Centre for Dentistry Amsterdam (ACTA), University of Amsterdam and Vrije Universiteit Amsterdam, 1081 LA Amsterdam, The Netherlands; 5 Department of Oral Kinesiology, Academic Centre of Dentistry Amsterdam (ACTA) University of Amsterdam and Vrije Universiteit Amsterdam, 1081 LA Amsterdam, The Netherlands; 6 Radboudumc 3DLab The Netherlands, Radboud University Medical Centre, Radboud Institute for Health Sciences, 6525 GA Nijmegen, The Netherlands

**Keywords:** proof-of-concept, navigation system, intraoperative navigation, real-time, feedback, temporomandibular joint prosthesis, maxillofacial surgery

## Abstract

**Background/Objectives**: One key objective in temporomandibular joint replacement is to precisely position the implant according to the virtual surgical plan, utilizing drilling and osteotomy guides for accuracy. However, implementing this process can be challenging, as—even though the drilling and osteotomy guides should only fit in one position—there often are still multiple potential positions for both guides and implants on smooth bony surfaces. Even minor deviations in the implant’s placement can affect wear, influence biomechanical behavior, and lead to adverse outcomes. Intraoperative navigation has emerged to verify the alignment of implants with the preoperatively planned ideal position. While the use of navigation systems in TMJ surgery is well documented for certain procedures, its application in TMJ replacement cases has been limited. **Methods**: In this study, two methods to improve the accuracy of TMJ replacement are introduced: a new marker-based navigation workflow and the use of orientation screws in two patients. **Results**: Unlike conventional navigation methods, the marker-based system provides a more intuitive method for assessing the 3D orientation of the TMJ implant concerning the planned position, enhancing surgical accuracy. The addition of a guiding screw provides a reference point to enhance the accuracy of guide placement. **Conclusions**: The accurate placement of the prosthesis largely relies on the precise positioning of the guides. Even slight inaccuracies in the position of the TMJ prosthesis, resulting from suboptimal guide placement, can lead to significant negative clinical outcomes. Marker-based navigation and the use of guiding screws may potentially improve the precision of TMJ replacement procedures.

## 1. Introduction

The restoration of function, facial symmetry, and relief from pain in conditions leading to pathological modifications of the temporomandibular joint (TMJ) sometimes necessitates the use of TMJ implants. In recent decades, significant strides have been made in TMJ implant design, with a particular focus on patient-specific solutions and innovative approaches to address individual anatomical variations [1,2,3,4,5]. The integration of virtual surgical planning and computer-aided design and manufacturing (CAD/CAM) has paved the way for diverse designs of both the fossa and mandibular components, ensuring a tailored and optimal fit for each patient [2,6].

Customized or patient-specific implants (PSIs) are accompanied by positioning guides during placement, aiming to replicate the meticulously planned position of the implant in the intraoperative setting. However, the practical implementation of this process often poses challenges, with multiple potential positions for guides and implants, especially on smooth bony surfaces [2]. Accurate placement of the prosthesis is primarily dependent on the precise placement of the positioning guides. It may be challenging to accurately place the positioning guide due to the absence of anatomical landmarks and the limited surgical exposure. Minor inaccuracies in TMJ prosthesis position, yielded by suboptimal placement of the positioning guide, can result in significant adverse clinical outcomes since the passive fit of the prosthesis may be affected.

Surgical precision is essential to accurately achieve the preoperatively predicted functional outcome, wear characteristics, and biomechanical behavior of the patient-specific prosthesis [7]. Discrepancies between the planned and postoperative positions of the prosthesis can impact its expected function and performance, potentially leading to malocclusions and facial asymmetry, or result in unexpected and increased loading conditions, and eventually, to implant failure and loss [8,9]. Another challenge with the condylar prosthesis is maintaining its position in the glenoid fossa, both immediately after surgery and during long-term use. Additionally, numerous reports in the literature document a variety of complications, such as persistent pain, erosion into the external auditory meatus, erosion into the middle cranial fossa, migration, fracture of the prosthesis, and plate exposure [10,11].

To address these challenges, intraoperative navigation emerges as a promising solution, providing a means to verify the alignment of the implant with the preoperatively planned ideal position [12,13,14,15,16]. While the application of navigation systems in TMJ surgery is well documented for certain procedures, such as gap arthroplasty and ankylosis resection, its utilization in TMJ replacement cases has been limited [2,17,18,19,20].

Navigation markers have been introduced as tools to enhance the intraoperative assessment of implant positioning using navigation systems [21]. Unlike traditional contour-based feedback provided in multiplanar views, this approach focuses on defined reference points integrated into the implant design. This method visualizes the relationship between the real-time position of the navigation pointer and the preoperatively planned position of the marker. Quantitative feedback is provided, measuring the three-dimensional deviation between the actual and preplanned marker positions. This system offers the surgeon more intuitive, three-dimensional feedback regarding the positioning of the implant [21].

To improve the accuracy of the condylar positioning guide, the use of guiding screws to provide an extra landmark is implemented in this study [22].

This paper introduces two methods for accuracy improvement in TMJ replacement. Firstly, a novel marker-based navigation workflow, presenting a proof-of-concept study that incorporates navigation feedback during implant insertion. Unlike traditional navigation approaches, the marker-based system offers enhanced interpretability of the three-dimensional implant positioning [16]. Drawing inspiration from successful applications in orbital reconstruction, our hypothesis is that this approach provides a more intuitive means of evaluating the 3D orientation of the TMJ implant in relation to the planned position, thereby offering potential improvements in surgical accuracy. Secondly, to enhance the accuracy of the workflow for the condylar positioning guide and prosthesis, guiding screws are introduced.

## 2. Materials and Methods

### 2.1. Study Design

A proof-of-concept study is reported describing and evaluating the utilization and accuracy of a marker-based navigation workflow for bilateral TMJ replacement in two patients with one- and six-month follow-up to ensure no recurrence and no postoperative complications. This case report was conducted in accordance with the Declaration of Helsinki. Informed written consent was obtained from the patients for scientific and photo publication.

### 2.2. Case Description

**Figure 1:**
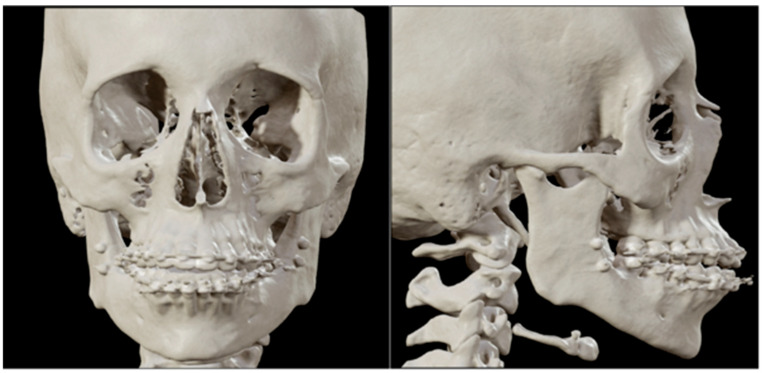
Patient 1: front and lateral preoperative 3D scan.

**Figure 2:**
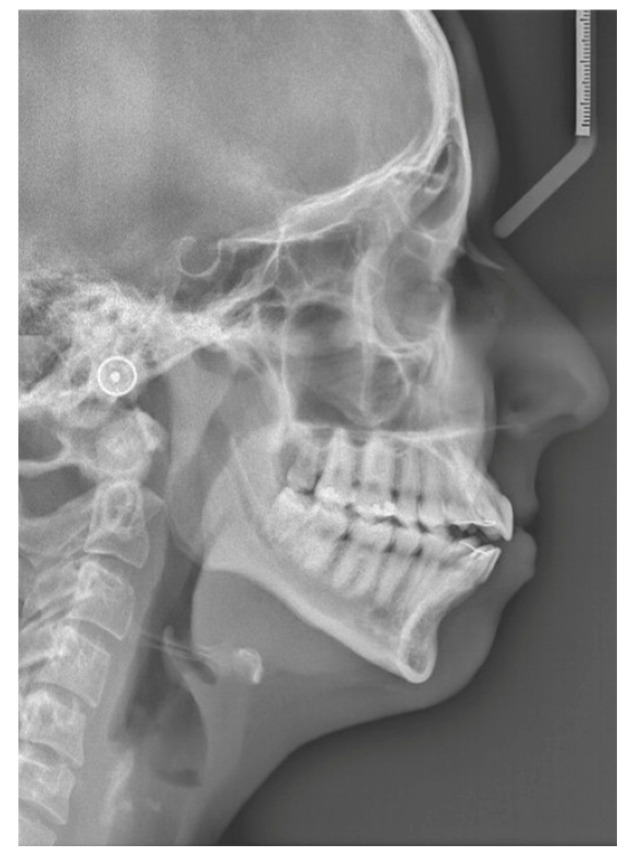
Patient 1: preoperative LSP. Clearly illustrating the anterior open bite.

The first patient is a 21-year-old woman with no relevant medical history and medication use, who presented to the Department of Oral and Maxillofacial Surgery and Head and Neck Oncology of the Amsterdam UMC—a tertiary referral university hospital—with facial pain and a progressive open bite. The maximum opening of the mouth (MMO) was 40 + 5 mm. She was diagnosed with progressive condylar resorption of the left and right temporomandibular joint. The preoperative three-dimensional (3D) scan and lateral cephalogram (LSP) showed flattening of the condyles on both sides, narrowed joint space, and atrophic changes of the condyles on both sides (Figure 1 and Figure 2).

**Figure 3:**
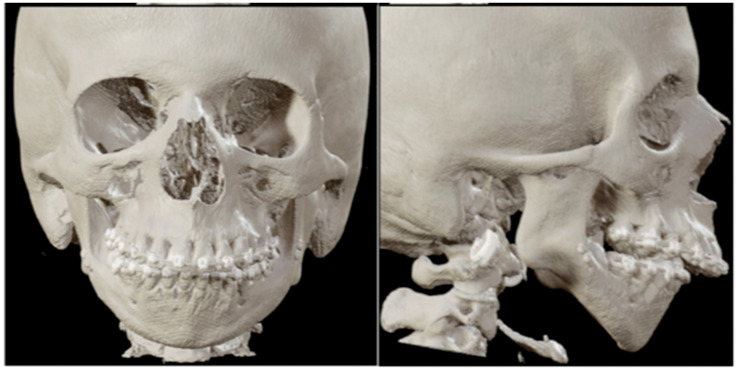
Patient 2: front and lateral preoperative 3D scan.

**Figure 4:**
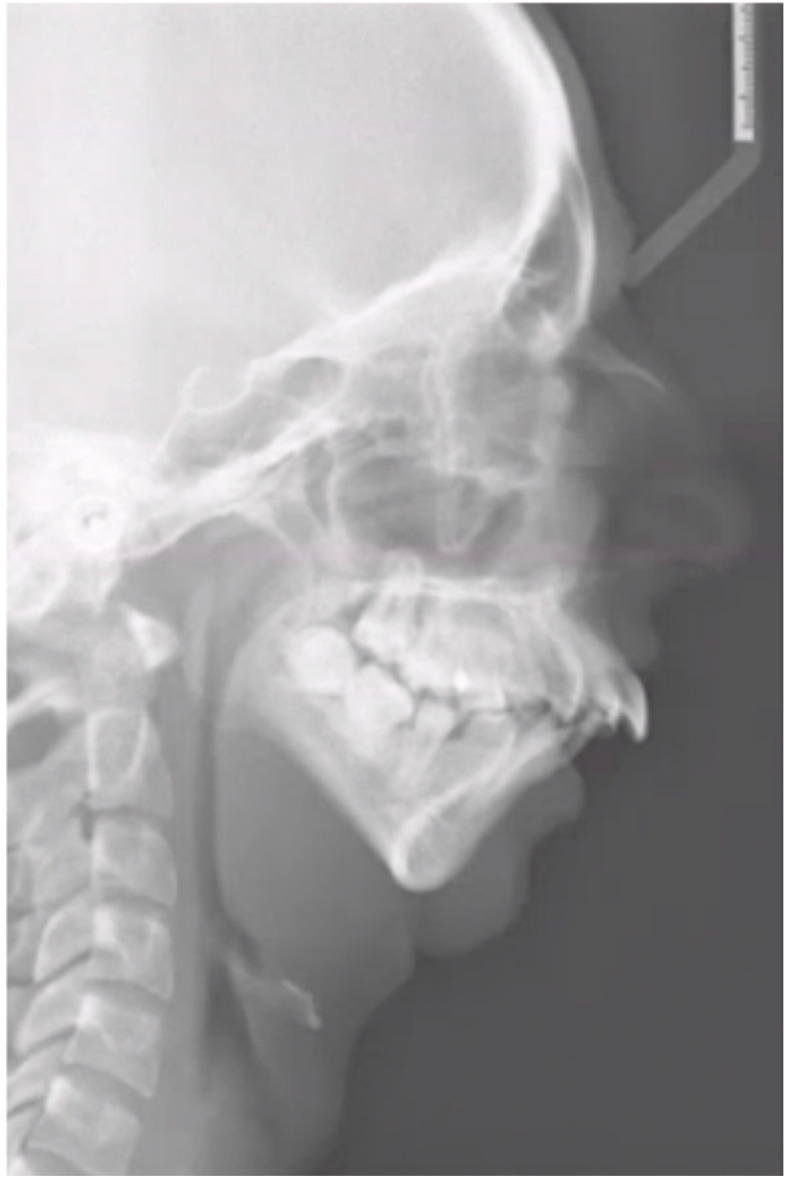
Patient 2: preoperative LSP showing atrophic temporomandibular joints, severe retrognathia, and a narrowed upper airway.

The second patient is a 19-year-old woman with a medical history of juvenile idiopathic arthritis and retrognathia due to growth restriction as a result of chronic arthritis. At the age of 13, the patient was treated with an intra-articular steroid injection in the left temporomandibular joint due to pain complaints. Five years later, the patient was diagnosed with severe condylar resorption of the left and right temporomandibular joint and dental and skeletal correction was indicated due to a class II malocclusion and severe retrognathia. The maximum opening of the mouth (MMO) was 27 mm. The preoperative 3D scan and LSP (Figure 3 and Figure 4) showed highly atrophic temporomandibular joints, severe retrognathia, and severe protrusion of the lower front.

### 2.3. Data Acquisition

Preceding the surgical procedure, the patients were treated with passive full dental arch orthodontic fixed appliances (brackets) to achieve stable arches. Additionally, in order to be able to use passive navigation for the mandibular component, four 5 mm orientating screws (KLS Martin 2.0 system, Tuttlingen, Germany) were placed under local anesthesia on the lateral side of the left and right mandibular ramus (two per side) [22]. A CT scan (Somatom Force, Siemens Healthineers, Forcheim, Germany) was performed using a standardized CT head scan protocol (patient 1: 90 kV, 100 mA, 223 × 223 mm FOV, 0.6 mm slice thickness, reconstruction kernel Hr 38 soft; patient 2: Sn150 kV, 250 mAs, 12.1 CTDI, 207 × 207 mm FOV, 1 mm slice thickness, reconstruction Kernel Hr 64). Additionally, an intraoral scan (Trios 3, 3Shape, Copenhagen, Denmark) was obtained to capture high-resolution dental information. This dataset was securely transmitted to the SurgiCase platform (Materialise NV, Leuven, Belgium) for virtual surgical planning and implant design.

### 2.4. Virtual Surgical Planning

**Figure 5:**
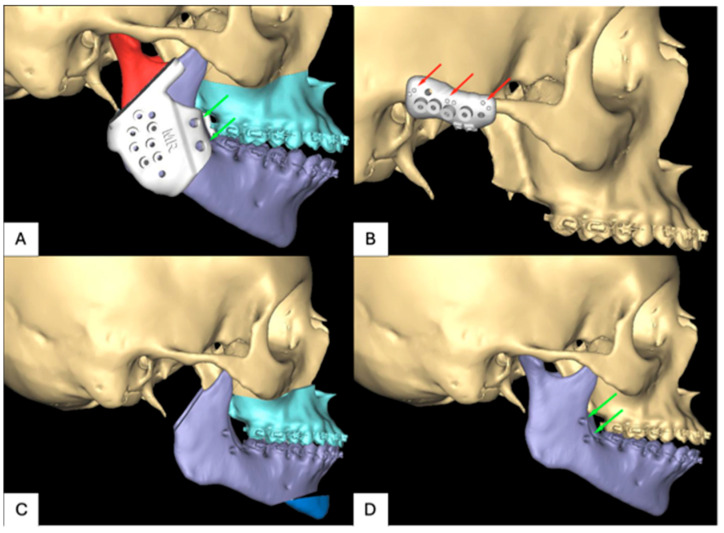
Virtual surgical planning for patient 1: (**A**) positioning screws, (**B**) navigation markers, (**C**) planned resection, and (**D**) preoperative side.

**Figure 6:**
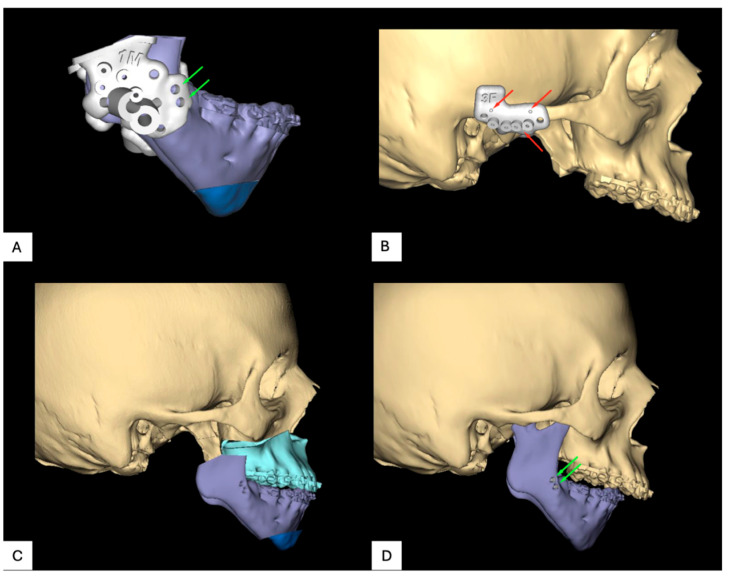
Virtual surgical planning for patient 2: (**A**) positioning screws, (**B**) navigation markers, (**C**) planned resection, and (**D**) preoperative side.

The virtual surgical planning (Figure 5 and Figure 6) was performed through virtual web meetings between the surgeon and the clinical engineer at Materialise. First, the maxilla and mandible were put in the desired end occlusion, and the complete maxillomandibular complex was moved to the desired final position. In order to improve the esthetic profile of the patient, a virtual genioplasty was planned in both cases for additional advancement of the chin. A cutting guide, incorporating the orientation screws in the mandibular ramus for passive navigation—in other words, positioning feedback—was designed for resection of the condylar process. This guide also served as a predrilling guide for the PSI mandibular component fixation screws. Osteotomy and positioning guides were designed for the genioplasty, whereas patient-specific titanium predrilling guides and implants were designed for the Le Fort osteotomy [23,24].

A separate predrilling guide for the fossa component, extending from the mastoid bone to the intact zygomatic arch, marked the fixation screw locations ventral and dorsal to the condylar fossa. Navigation markers were incorporated into this guide to facilitate marker-based navigation feedback for positioning control [21].

### 2.5. Implant Design

**Figure 7:**
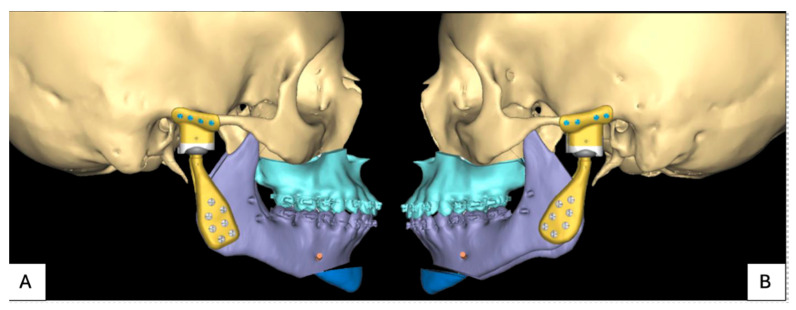
PSI for patient 1: (**A**) planned position implants right and (**B**) planned position implants left.

**Figure 8:**
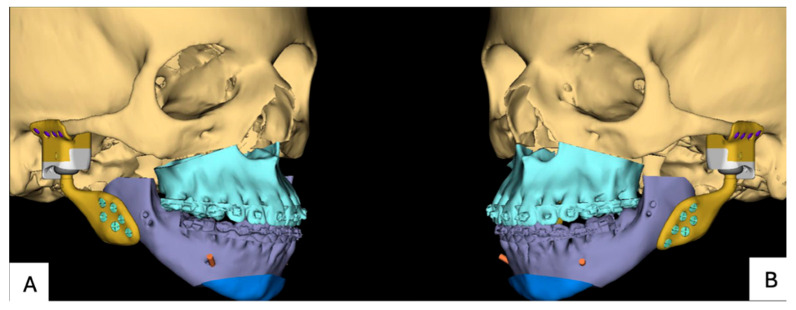
PSI for patient 2: (**A**) planned position implants right and (**B**) planned position implants left.

The patient-specific implants (PSIs) were designed through virtual web meetings between the surgeon and the clinical engineer at Materialise. The fossa component and mandibular component incorporated the predrilled holes in the zygoma, mastoid, and mandibular ramus (Figure 7 and Figure 8). Notably, in the fossa component, a navigation landmark was designated at the head of the screw, connecting the ultra-high-molecular-weight polyethylene (UHMWPE) and titanium components. This deliberate marking facilitated marker-based navigation feedback during the reconstruction stage.

### 2.6. Surgery

In both cases, the surgery was performed by two oral and maxillofacial surgeons—both specialized in TMJ reconstruction. The procedure encompassed the resection of the left and right temporomandibular joints, the Le Fort 1 osteotomy and genioplasty.

**Figure 9:**
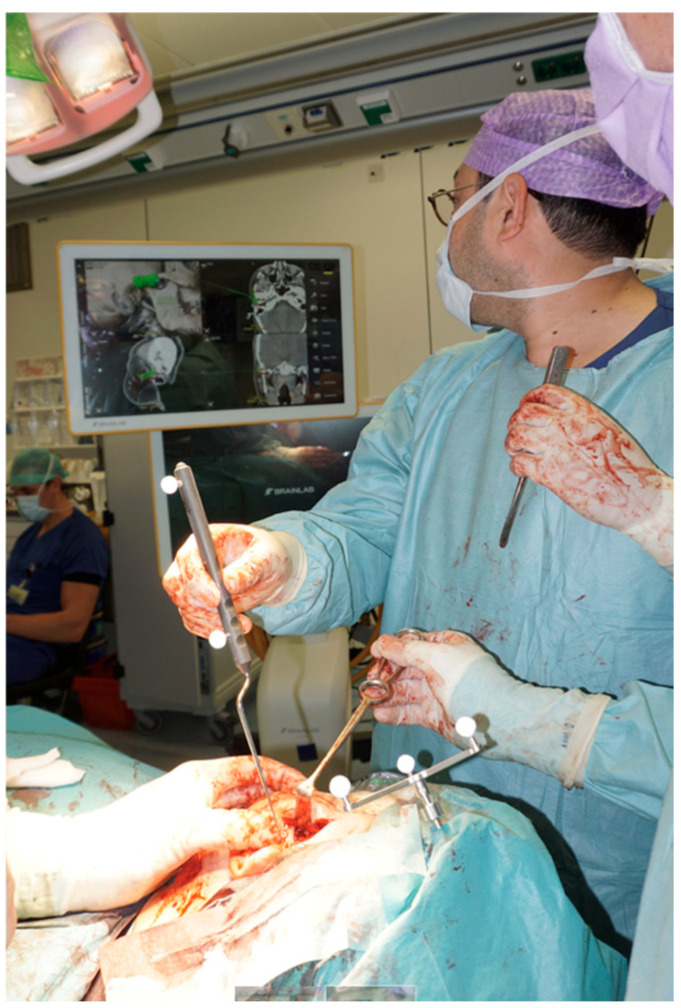
Navigation set-up.

Firstly, the TMJ was completely resected, including the condylar process of the mandible, synovial tissue, TMJ capsule, articular disk of the TMJ, lateral pterygoid muscle, articular surface of the temporal bone, temporomandibular ligament, and sphenomandibular ligament. The abdominal fat graft was harvested. Subsequently, a customized total joint prosthesis was precisely placed. After the placement of the prosthesis and the abdominal fat graft, the Le Fort 1 osteotomy and genioplasty were performed. Intraoperative navigation (Brainlab Curve, optical navigation system) played a pivotal role in verifying the accurate placement of the total joint prosthesis. The navigation set-up is shown in Figure 9. The camera was positioned at the head of the operating table. A reference array was fixed to the left side of the cranium, thereby providing a stable reference point for positional tracking throughout the navigation process. To verify the accuracy of the registration, several anatomical landmarks were checked using a navigated pointer. These landmarks included the forehead, the nasion, both medial and lateral canthi bilaterally, as well as the tragus on both sides. During surgery, the navigation pointer was used to align the implant with its preoperatively planned position. The system provided quantitative feedback on the spatial deviation between the location of the implant and its preoperatively planned position, allowing the surgeon to make adjustments. Guided by the navigation system, the surgical procedure was carried out. There were no intraoperative complications or adjustments due to navigation.

### 2.7. Positioning Accuracy Quantification

**Figure 10:**
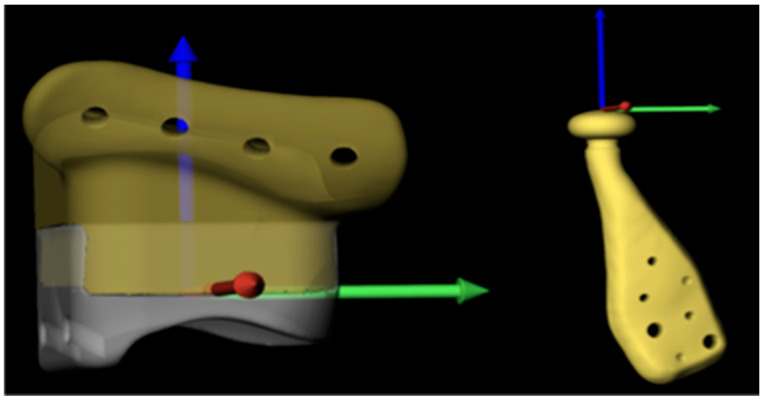
Reference frame for the fossa component and mandibular component. The *x*-axis: ventrodorsal direction of the UHMWPE component, the *y*-axis: mediolateral direction and the *z*-axis: caudocranial direction.

A reference frame was created for the left and right stereolithographic models (STL) of the UHMWPE fossa components’ postoperative positioning analysis in Blender (v4.0, Blender Foundation, Amsterdam, the Netherlands). The deepest point of the articulating surface was calculated and used as the origin. The x-axis aligned with the ventrodorsal direction of the UHMWPE component, the y-axis with the mediolateral direction and the z-axis with the caudocranial direction (see Figure 10). Since only a combined UHMWPE + titanium virtual model of the fossa component was available, the reference UHMWPE component was matched on the patient’s planned fossa component. The UHMWPE component was subtracted using a Boolean operation to obtain the titanium fossa component. After surgery, a second CT scan (Discovery CT750 HD, GE Medical Systems, Milwaukee, WI, United States) was acquired using the following scan parameters: patient 1: 120 kV, 50 mA, 6.54 CTDI, 180 × 180 mm FOV, 1 mm slice thickness mpr reconstruction, reconstruction kernel BONEPLUS; patient 2: 120 kV, 50 mA, 6.54 CDTI, 194 × 194 mm FOV, 1 mm slice thickness mpr reconstruction, reconstruction kernel (BONEPLUS). Segmentations of the postoperative mandibular components and titanium fossa components were created in Brainlab software (Origin version 3.4), using a 2000 Hounsfield Unit (HU) threshold, and exported in STL format.

In 3DMedX (Radboudumc, Nijmegen, the Netherlands), the preoperative CT scan was imported, along with the virtual planning models of the fossa titanium components, fossa UHMWPE components, and mandibular components, and the planned position of the mandible. The reference UHMWPE components were imported, and the postoperative CT data and postoperative segmentations were imported. A voxel-based matching, using the cranial base as the region of interest, was performed to align the postoperative data with the preoperative data. The iterative closest point (ICP) approach was subsequently used to move the planned position of each UHMWPE fossa into the reference position Tplan -> ref. The same transformation was applied to the segmentation of the ipsilateral postoperative fossa component. The planned model (in the reference position) was matched based on the (transformed) postoperative segmentation (again using an ICP approach) to obtain the deviation in the x-direction, y-direction, and z-direction of the fossa reference frame (Figure 10).

To eliminate the effect of the mandible position on the quantitative outcome for the mandibular components, a threshold-based segmentation (360 HU) was generated of the postoperative mandible in 3DMedX. The resulting virtual model was matched based on the preoperative planned position of the mandible using an iterative closest point approach. The resulting transformation was applied to the postoperative mandibular component segmentation. The mandibular component and postoperative segmentation were brought to the reference position through the transformation with Tplan -> ref; an additional translation of 0.3 mm in the z-direction was added to correct for the gap between the fossa and the condylar head and align the top of the condylar head with the origin. The deviation in the x-direction, y-direction, and z-direction was obtained similar to the workflow for the fossa components of the planned components.

## 3. Results

### 3.1. Patient Outcome

**Figure 11:**
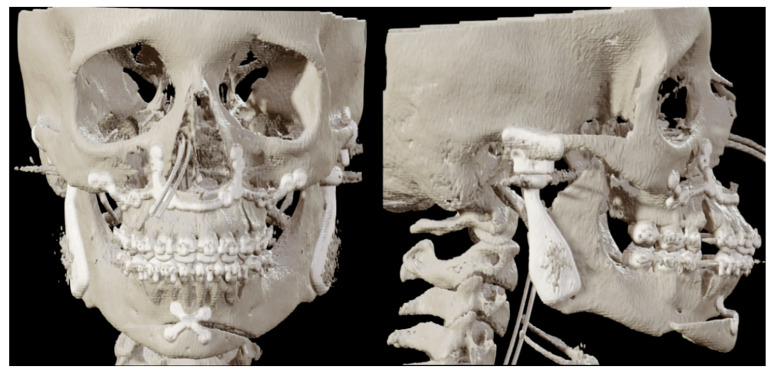
Patient 1: front and lateral postoperative 3D scan with the PSI in place.

**Figure 12:**
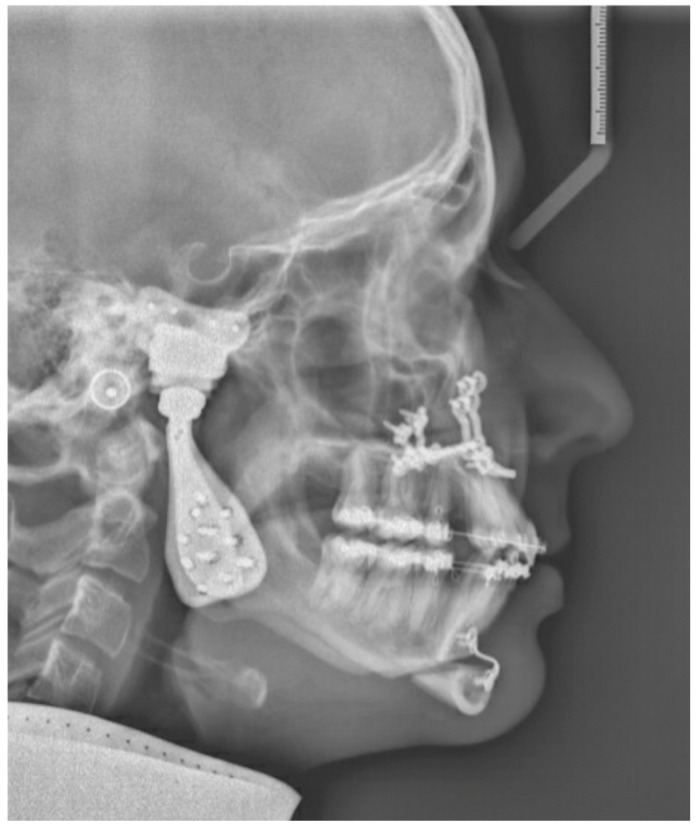
Patient 1: LSP two months post-TMJ reconstruction, Le Fort 1 osteotomy and genioplasty.

Two months post-surgery, the first patient reported notable improvements in pain relief. Food intake posed no challenges, and the temporomandibular joint exhibited satisfactory rotation and translation. The MMO improved to 40 + 4 mm after eight months. Figure 11 shows the postoperative 3D scan. Figure 12 shows the LSP two months post-surgery.

**Figure 13:**
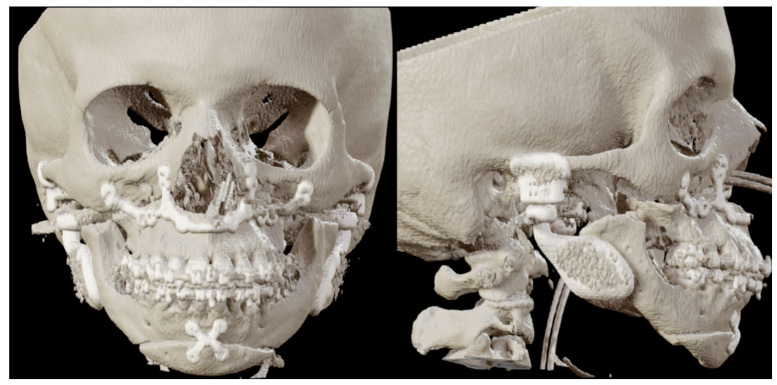
Patient 2: front and lateral postoperative 3D scan with the PSI in place.

**Figure 14:**
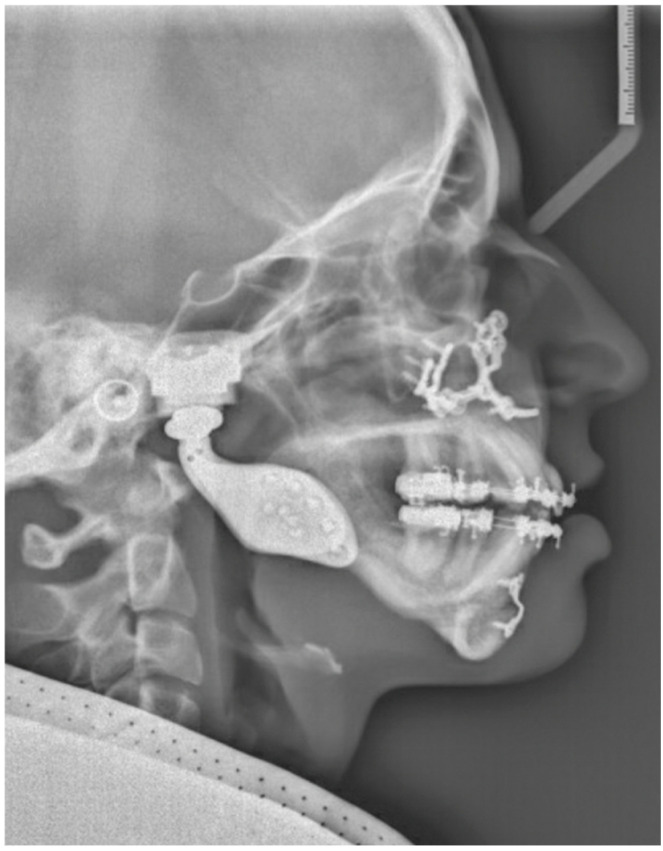
Patient 2: LSP two months post-TMJ reconstruction, Le Fort 1 osteotomy and genioplasty.

The second patient reported no pain and an improvement in aesthetics one month post-surgery. Figure 13 shows the postoperative 3D scan. Figure 14 shows the LSP one-month post-surgery. There was a stable occlusion and the MMO was 55 + 6 mm. Food intake posed some challenges but was improving.

### 3.2. Reconstruction Accuracy

The quantitative positioning outcomes for patients 1 and 2 are provided in Table 1. The mean Euclidean distance between the planned and obtained fossa was 1.5 mm; for the mandible, the mean distance between the planned and obtained condyle was 1.8 mm.

## 4. Discussion

The navigation system enables the surgeon to visualize the real-time position of surgical instruments or implants on a monitor displaying the patient’s CT or MRI 3D data. These systems integrate imaging with the surgical field, allowing simultaneous visualization of different image types to reveal structures usually visible only intraoperatively and permitting navigation in anatomically sensitive areas. Navigation systems are now commonly used in craniomaxillofacial surgery due to their reliability and accuracy, typically within 1–2 mm [25,26,27,28]. Over the years, these systems have evolved and simplified the surgical procedure by minimizing the intraoperative invasiveness. The development of navigation-assisted surgery has improved execution and predictability, enabling greater precision in oral and maxillofacial procedures [29,30]. Setting up a navigation system takes an experienced team approximately 5–10 min, and an additional 10 to 20 min are required for result verification. The accuracy of navigation systems is limited by the specific system used, the method of obtaining imaging data, and the synchronization of this data with the patient’s actual position during the procedure [29].

Navigation-assisted surgery remains challenging because the surgeon focuses on two-dimensional multiplanar views in relation to preoperative planning. This study incorporated navigation markers in the TMJ replacement implant design and indicated in the surgical planning. Intraoperatively, feedback can be obtained by positioning the pointer in these fixed marker points [31]. Possible advantages of this method include more intuitive and quantitative feedback during reconstruction; in studies on orbital reconstruction, the marker-based method demonstrated improved reconstruction accuracy compared to conventional surgical navigation. This feedback method also proved feasible in TMJ reconstruction.

The mobile nature of the mandible complicates synchronization with preoperative imaging data and makes accurate use of surgical navigation infeasible. The use of a 3D-printed predrilling and resection guide facilitates transfer of the virtual surgical plan to the operation room and reduces the risk of inaccurate prosthesis positioning. However, accurately positioning these guides may be difficult due to the lack of anatomical landmarks and limited visibility. In this paper, the addition of the guiding screw provides a reference point to improve the accuracy of the placement of the guide. A pilot study by Ho et al. illustrated the use of guiding screws in edentulous patients requiring orthognathic surgery [22]. On comparison of the planned and achieved movements of the maxilla and mandible, the study demonstrated this method to be accurate and predictable.

Whereas most TMJ replacement surgery is performed without navigation, musculoskeletal modeling studies have demonstrated that implant malposition elevates stress on system components and thereby increases the risk of mechanical failure due to early screw loosening, screw fracture, implant loosening, and implant displacement [32]. These issues contribute to approximately 16% of postoperative complications in TMJ replacement surgery [33]. This paper introduces a new method for assessment of surgical accuracy in TMJ reconstruction. There are several advantages to the method: it is observer-independent and quantifies the surgical result in all degrees of freedom. Since the positions of the interacting surfaces of the condyle and fossa components are expected to affect the function of the reconstructed joint, the analysis method takes these landmarks as the origin position for calculating the discrepancy between planning and the surgical result. The outcome of the evaluation is a distance measurement that is easily interpretable; differences in the size and shape of implants between patients do not affect the outcome of the measurement. A possible drawback of this method might be that an overall inaccurately positioned implant may still have an acceptable deviation at the interacting surface of the condyle and fossa component, but in the authors’ opinion, the benefits of a simple and interpretable method that could relate to the patient’s outcome outweigh this drawback. It is expected that the accuracy of the navigation system will play a key role in improving the function of the implant, but further clinical research is needed to determine the direct impact of this accuracy on patient-centered outcomes, such as pain reduction and functional improvement.

## 5. Conclusions

This proof-of-concept study introduces two methods to improve accuracy in TMJ replacement. The first method is a novel marker-based navigation workflow, presenting two cases that incorporated navigation feedback during implant insertion. The preoperative data were aligned to the postoperative data and demonstrated that the marker-based navigation method leads to an overall accurate placement of the PSI and TMJ implant. The second method entails the addition of the guiding screw and provides a reference point to improve the accuracy of the placement of the guide. Marker-based navigation and the use of guiding screws can possibly improve accuracy in TMJ replacement. There is a need for research that systematically compares navigated and non-navigated methods, incorporating both time and cost factors into the analysis.

## Figures and Tables

**Table 1:** Implant position in implant-oriented navigation.

	X (mm)	Y (mm)	Z (mm)	Euclidean (mm)
Patient 1				
Fossa right	0.9	−1.1	−0.2	1.5
Fossa left	−0.6	−2.6	−1.5	3.1
Condyle right	−1.1	0.5	0.8	1.5
Conduyle left	0.9	0.7	2.0	2.3
Patient 2				
Fossa right	0.4	−0.1	0.3	0.5
Fossa left	1.0	0.4	−0.2	1.1
Condyle right	−0.3	−0.4	0.9	1.1
Condyle left	−1.5	−0.1	1.8	2.3

## Data Availability

The data presented in this study are available on request from the corresponding author.
